# A novel aerosol collection method shows the cough aeromicrobiome of people with tuberculosis is phylogenetically distinct from respiratory tract specimens

**DOI:** 10.21203/rs.3.rs-4106141/v1

**Published:** 2024-04-11

**Authors:** Tinaye L. Chiyaka, Georgina R. Nyawo, Charissa Naidoo, Suventha Moodley, Jose C. Clemente, Stephanus T. Malherbe, Robin Warren, David Ku, Leopoldo N. Segal, Grant Theron

**Affiliations:** Stellenbosch University; Stellenbosch University; Stellenbosch University; Stellenbosch University; Icahn School of Medicine at Mount Sinai; Stellenbosch University; Stellenbosch University; Georgia Institute of Technology; New York University School of Medicine; Stellenbosch University

**Keywords:** PneumoniaCheck, Tuberculosis, Microbiota, Aerosols, Xpert MTB/RIF Ultra

## Abstract

**Background:**

Tuberculosis (TB), a major cause of disease and antimicrobial resistance, is spread via aerosols. Aerosols have diagnostic potential and airborne-microbes other than *Mycobacterium tuberculosis complex* (MTBC) may influence transmission. We evaluated whether PneumoniaCheck (PMC), a commercial aerosol collection device, captures MTBC and the aeromicrobiome of people with TB.

**Methods:**

PMC was done in sputum culture-positive people (≥30 forced coughs each, n=16) pre-treatment and PMC air reservoir (bag, corresponding to upper airways) and filter (lower airways) washes underwent Xpert MTB/RIF Ultra (Ultra) and 16S rRNA gene sequencing (sequencing also done on sputum). In a subset (n=6), PMC microbiota (bag, filter) was compared to oral washes and bronchoalveolar lavage fluid (BALF).

**Findings:**

54% (7/13) bags and 46% (6/14) filters were Ultra-positive. Sequencing read counts and microbial diversity did not differ across bags, filters, and sputum. However, microbial composition in bags (*Sphingobium-, Corynebacterium-, Novosphingobium-enriched*) and filters (*Mycobacterium-, Sphingobium-, Corynebacterium-enriched*) each differed vs. sputum. Furthermore, sequencing only detected *Mycobacterium* in bags and filters but not sputum. In the subset, bag and filter microbial diversity did not differ vs. oral washes or BALF but microbial composition differed. Bags vs. BALF were *Sphingobium*-enriched and *Mycobacterium-, Streptococcus*-, and *Anaerosinus*-depleted (*Anaerosinus* also depleted in filters vs. BALF). Compared to BALF, none of the aerosol-enriched taxa were enriched in oral washes or sputum.

**Interpretation:**

PMC captures aerosols with Ultra-detectable MTBC and MTBC is more detectable in aerosols than sputum by sequencing. The aeromicrobiome is distinct from sputum, oral washes and BALF and contains differentially-enriched lower respiratory tract microbes.

## Background

Tuberculosis (TB) is a serious global health concern, with an estimated 10.6 million cases and 1.3 million fatalities in 2022^1^. Not only does TB remain challenging to diagnose with an urgent need for non-sputum based tests but the characteristics of cough aerosols from people with TB, which are a determinant of transmission success, are poorly undefirstood^2,3^.

Breath-based detection of *Mycobacterium tuberculosis complex* (MTBC) DNA is a promising non-sputum method for diagnosing TB, with face masks and blow tubes under evaluation^4–6^. PneumoniaCheck (PMC), a cough aerosol collection device, is evaluated in people with cystic fibrosis (CF)^7^ and viral pneumonia^8^. In the first study, CF-related bacteria were in the aerosols of 65% of people sputum-positive for these bacteria (and aerosol did not contain lung commensals found in sputum). In the second study, when pathogen readouts from PMC and bronchoalveolar lavage fluid (BALF) were compared, 66% of aerosols were PCR-positive. PMC, is however, unevaluated in TB where, in addition to detecting MTBC in exhaled aerosols, it could be used to characterise the aeromicrobiome, which may influence contacts’ immune responses.

The microbiota is a topic of increasing interest in TB, where sputum is widely studied. However, the sputum microbiota more closely resembles the upper respiratory tract (URT) than the lower respiratory tract (LRT)^9,10^, which is the primary site-of-disease in TB. Sampling the LRT is difficult because it requires bronchoscopy^11^, which is invasive, ethically complex for research purposes only, and expensive, often rendering which unfeasible in large cohorts where TB is prevalent^12^. Aerosols, which are more accessible, could be a useful proxy for studying the LRT, as aerosols partly originate from the LRT^13^.

PMC comprises a 250mL air reservoir (bag) attached to a mouthpiece with a filter. PMC is designed to separate aerosols from the URT and LRT into the bag and filter, respectively^14^. This separation occurs after a person coughs into the PMC, at which point air from the anatomical URT dead space (~ 150mL)^15^ flows first into the bag. Air after the 150mL is likely from the LRT and then, due to backpressure from the inelastic bag, directed towards the filter^7^.

It is thus possible that, in addition to PMC-captured aerosols being useful for TB diagnosis, such aerosols may serve as an alternative to BALF for LRT microbiota characterization and more accurately represent the vehicle of TB transmission, including compared to oral washes and sputum. We therefore evaluated MTBC detection by Xpert MTB/RIF Ultra (Ultra) and the bag and filter microbiota. We compared microbiota in the bag and filter to URT and LRT clinical samples.

## Methods

### Ethics

Stellenbosch University Health Research Ethics Committee (SU-HREC) approved the study (N14/10/136, N16/05/070). People provided a written informed consent.

### Recruitment and data collection

This study involved people (n = 16; ≥ 18 years) enrolled at primary healthcare facilities in Cape Town. People were sputum MTBC culture-positive and not on treatment. Clinical and demographic data were collected.

### Sample collection

**Figure 1:**
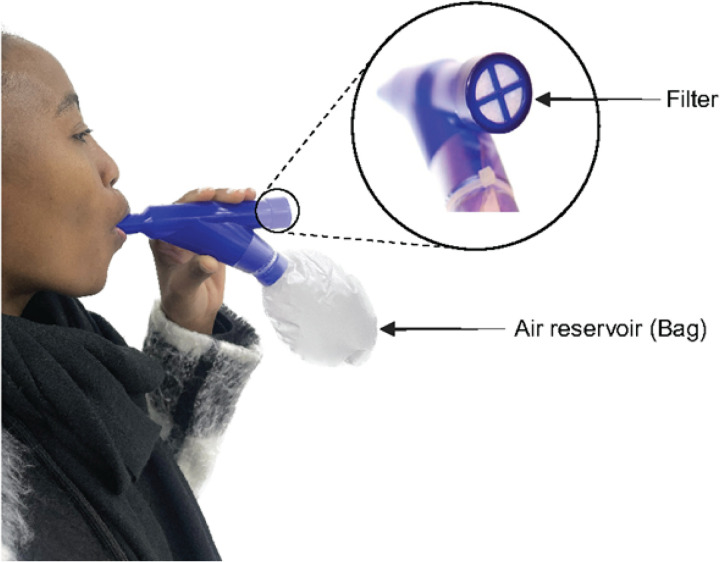
How to use pneumonia check (PMC): People seal their lips around the mouthpiece ridge and cough into the PMC. As they exhale, air from the anatomical dead space moves into the inelastic air reservoir (bag), while additional air, primarily from the lower respiratory tract (LRT) is directed towards the filter.

People first gave aerosols collected using PMC and sputum was then induced. For aerosols, people were asked to take deep breaths to stimulate a cough. While sealing lips around the PMC mouthpiece ridge, with teeth rested on the device notches, people were asked to produce ≥ 30 deep coughs (Fig. 1). Bags were deflated by hand squeezing after each cough. People who had adverse effects like dizziness could rest between coughs and, if they produce sputum during coughing, were given a jar in which to expectorate. DNA sampling background controls (BKG) were collected to identify potentially contaminating taxa and included an unused PMC handled in the same manner as those used by people. Sputum was induced with 5% saline for 10min. Oral washes (representing URT) and BALF (representing LRT) were also collected in a subset (n = 6) of the 16 people (**Supplementary Methods**). This subset had been enrolled into a separate study examining site-of-disease immunological signatures (NCT03350048).

### Respiratory fluids processing

Oral washes and sputum were decontaminated using N-acetyl-L-cysteine (NALC), pelleted (3,217x*g*), resuspended in 2mL PB (pH 6.8; BD, South Africa) and stored at −80°C^16^. 1mL raw BALF aliquots were stored at −80°C for microbiota analysis. MTBC in sputum was detected using the Xpert MTB/RIF (Xpert) and *Mycobacteria* Growth Indicator Tube (MGIT) 960 liquid culture according to the manufacturers’ methods.

### Recovery and processing of aerosols

Aerosols were separately recovered from the PMC bag and filter. After removal under sterile conditions, bags were rinsed and incubated for 15min in 10mL stripping buffer (1% Triton X-100 in 10mM Tris-HCl, pH 8.0) whereas the filter was submerged in 10mL stripping buffer, vortexed vigorously for one minute, and incubated for 30min. Bag and filter washes were pelleted (3,217x*g*) and resuspended in 1.5mL PB. Aliquots of 0.7mL each for MTBC testing using Ultra (version 2, according to the manufacturers’ methods) and microbiota analysis were stored at −80°C.

### 16S rRNA gene sequencing and analysis

Microbial DNA was extracted using the QIAamp DNA Mini kit (QIAGEN, Hilden, Germany). 16S rRNA gene sequencing (V4 region, 150 bp read length, paired end) was done on Illumina MiSeq platform^16,17^. Sequences were analysed using QIIME 2–2020.2^18^. We included samples with a minimum read count of 1000. Reads were clustered into amplicon sequence variants (ASVs) using *DADA2* (version 1.1.6)^19^ and taxonomy assigned at 99% similarity against GreenGenes^20^. α-Diversity (Shannon index) and β-diversity (Bray-Curtis dissimilarity index) were calculated using *vegan*^21^. Non-parametric methods (Mann-Whitney, Wilcoxon, Kruskal-Wallis, or Friedman tests for unpaired and paired comparisons) were used, whilst permutational multivariate analysis of variance (PERMANOVA) was used for β-diversity. *DESeq2*^22^ was used for differential abundance analyses with Benjamini-Hochberg multiple comparison adjustment. Potentially contaminating background taxa were identified using *decontam* (version 1.14.0)^23^. Taxa identified as possible contaminants were not removed from downstream analysis but greyed-out in volcano plots only if identified as differential. Data and R scripts are publicly available here.

## Results

### Study population

We included 16 people of median (IQR) age of 35 (25, 46) years. 31% (5/16) were HIV-positive, 75% (12/16) tobacco smokers, and 94% (15/16) of mixed ancestry (Table 1). Median (IQR) days to culture-positivity (TTP) was 5 (5, 7).

### Ultra on aerosols

Ultra sensitivity on bags and filters washes was 54% (7/13) and 43% (6/14, p = 0.568), respectively (Table 2). No sputum culture TTP differences occurred when compared based on the bag or filter Ultra results nor were there differences in Ultra-generated IS1081-IS6110 C_T_s. Ultra-generated sample processing control (SPC) cycle threshold (C_T_) values, a measure of PCR inhibition^24^, were similar when across bag and filter washes and when compared across people who had Ultra-positive vs. -negative bags or Ultra-positive vs. -negative filters.

### A distinct aeromicrobiome is detectable over and above background aerosol

The number of reads did not differ in sputum, bags, and filters in per person comparisons (nor in people who also had oral washes and BALFs sequenced) (**Figure S1a-b**). Given aerosols have low microbial biomass, we compared the microbiota in aerosol-exposed bags and filters to BKG to check for contamination. α-Diversity was similar between BKG and aerosols (p = 0.430, **Figure S2a**), but β-diversity differed (PERMANOVA; p = 0.001, **Figure S2b**), with some bag and filter samples grouping with BKG. Potential bag- and filter-contaminating taxa included *Pseudomonas-, Anaerosinus-, Alistipes* and-*Kocuria* (full list in **Table S1, Figure S3**), however, none of these were differential in other analyses.

### Compared to sputum, the aeromicrobiome differs and Mycobacterium is more detectable in the filter

**Figure 2:**
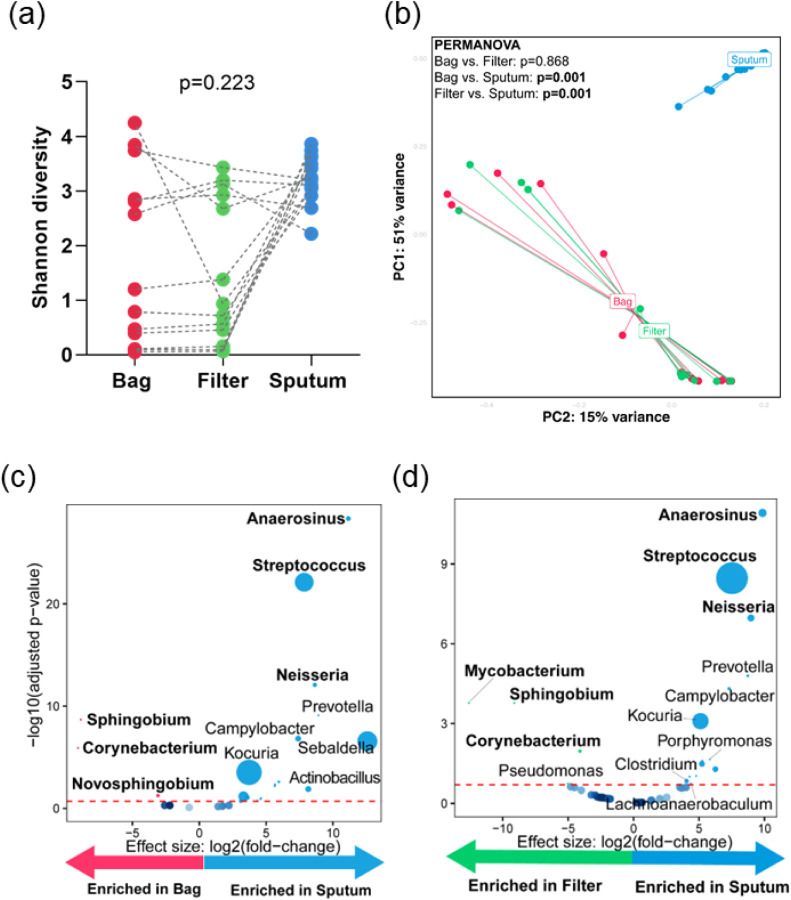
Aerosol microbiota is compositionally distinct from sputum: (a) paired α-diversity (Shannon index) using Friedman test for bag, filter, and sputum. (b) β-diversity (Bray-Curtis dissimilarity index), shows distinct clustering of sputum and aerosols. Volcano plots showing differentially abundant taxa in (c) bag vs. sputum, and (d) filter vs. sputum. Taxa that are considered discriminatory appear above threshold (marked by the red dotted line, FDR=0.2). The size of the dots corresponds to the relative abundance of the taxa.

Aerosols vs. sputum: α-Diversity (p = 0.223) was similar yet β-diversity (PERMANOVA; p = 0.001) differed (Fig. 2a–b). Aerosols were compositionally distant from sputum (**Figure S4a-b**). Bags were *Sphingobium-, Corynebacterium*- and *Novosphingobium*-enriched, and *Anaerosinus-, Streptococcus*- and *Neisseria*-depleted vs. sputum (Fig. 2c). Fifilters were *Mycobacterium-, Sphingobium*- and *Corynebacterium*-enriched, and *Anaerosinus-, Streptococcus*- and *Neisseria*-depleted vs. sputum (Fig. 2d).

Bags vs. filters: α-Diversity and β-diversity (p = 0.223 and PERMANOVA; p = 0.868) were similar between filters and bags (Fig. 2a–b) and differential abundance analyses showed no differential taxa (data not shown).

### The aeromicrobiome is not comparable to oral wash nor BALF

**Figure 3:**
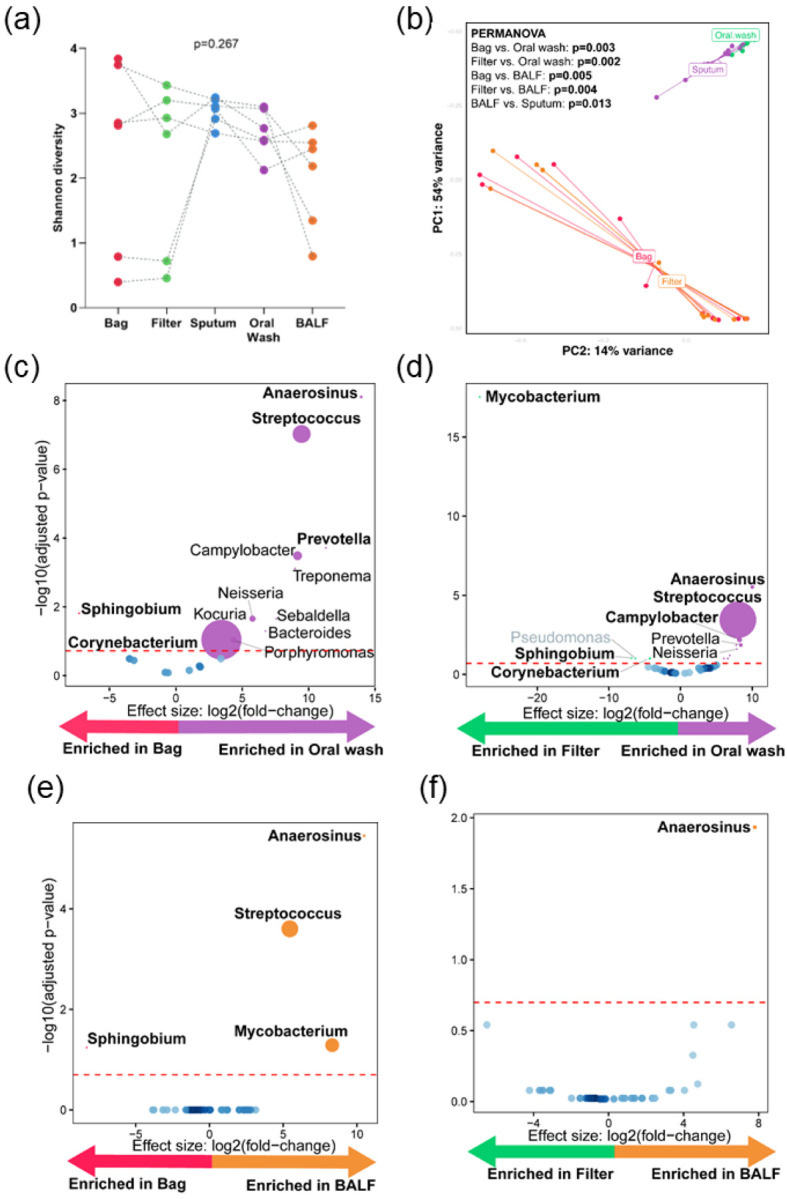
Aerosol microbiota neither similar to oral wash nor bronchoalveolar lavage fluid (BALF): (a) Paired α-diversity (Shannon index) using Friedmann test for bag, filter, sputum oral washes, and BALF (b) β-diversity (Bray-Curtis dissimilarity index) showing distinct clustering patterns for aerosols, sputum, oral washes and BALF. Volcano plots showing differentially abundant taxa in (c) bags vs. oral wash, (d) filters vs. oral wash, (e) bags vs. BALF, and (f) filters vs. BALF. Taxa that are considered discriminatory appear above threshold (marked by the red dotted line, FDR=0.2). The size of the dots corresponds to the relative abundance of the taxa. Taxa identified as potential contaminants are depicted in grey in the volcano plots.

α-Diversity was similar in aerosols and across respiratory fluids (p = 0.267, Fig. 3a).

Aerosols vs. oral wash: Oral wash β-diversity differed from bags and filters (PERMANOVA p = 0.005 and 0.002, respectively; Fig. 3b). Aerosols were compositionally different to oral washes (**Figure S4c**) with bags Sphingobium- and Corynebacterium-enriched and Anaerosinus-, Streptococcus- and Prevotella-depleted (Fig. 3c) and filters *Mycobacterium-, Pseudomonas*- and *Sphingobium*-enriched and *Anaerosinus-, Streptococcus*- and *Campylobacter*-depleted (Fig. 3d).

Aerosols vs. BALF: BALF β-diversity differed from bags and filters (PERMANOVA; p = 0.005 and 0.004 respectively, Fig. 3b). Aerosols were compositionally different to BALF (**Figure S4d**) with bags *Sphingobium*-enriched and *Anaerosinus-, Streptococcus*- and *Mycobacterium*-depleted and filters *Anaerosinus*-depleted (Fig. 3e–f).

### Differential abundance across sputum, oral wash, and bronchoalveolar lavage fluid

In BALF vs. sputum, *Mycobacterium*- was enriched and *Campylobacter*-, *Sebaldella*-, and *Prevotella*-depleted. In BALF vs. oral wash, *Mycobacterium*- was enriched and *Prevotella-, Campylobacter*-, and *Treponema*-depleted (**Figure S5a-b**).

## Discussion

We evaluated detection of MTBC and aeromicrobiome captured by PMC in people with TB. Our data shows 1) MTBC is detectable by Ultra in aerosols in about 57% of TB-positive people with comparable sensitivity in bags and filters, 2) bag and filter microbiota are compositionally similar, but differ to a similar extent vs. sputum, oral wash and BALF; and 3) the filter captures *Mycobacterium* more readily sequenced than in sputum and *Mycobacterium* itself is comparatively overrepresented in the aeromicrobiome vs. sputum. These findings show proof-of-concept for a novel sampling method for evaluating the aeromicrobiome, which we show is phylogenetically different from the microbiota in other respiratory fluids of people with TB.

The PMC bags and filters retained aerosols containing MTBC detectable using the WHO-recommended molecular test Ultra. This is consistent with studies that show PMC to capture LRT microbes in other diseases^7,8^. We did not detect differences in mycobacterial load nor PCR inhibition when comparing bags and filters, suggesting both PMC components may be useful, however, further optimization of aerosol collection procedures (e.g., number of coughs sufficient) and processing (to optimise release of captured material) require future investigation.

Diversity and composition metrics (α- and β-diversity) were similar between bag, filters, URT and LRT specimens; suggest a potential lack of separation of PMC-collected aerosol from these anatomical sites. As the primary purpose of the bag is to collect URT aerosols (~ 150mL in typical adults) but the volumetric capacity of the bag is 250mL, it is conceivable the bag retains aerosols originating from LRT in addition to URT, resulting in a mixture of LRT and URT microbiota.

The aeromicrobiome differed from sputum, oral wash, BALF microbiota, with aerosol depleted of anaerobic taxa such as *Prevotella* and *Streptococcus*, previously described as enriched in TB patients’ sputa and oral washes^25^ and highlighting that abundance in respiratory fluids does not necessarily translate into abundance in aerosol (previously described only for MTBC^26^). *Mycobacterium* was more detectable by sequencing in filter-captured aerosol than sputum, where sequencing this genus can be challenging even in people with severe pulmonary disease (TB and non-TB mycobacterial disease)^10,16^.This agrees with a prior study that did sequencing on mask-captured aerosol from people with TB^27^. Collectively, these findings suggests that, amongst the respiratory flora in people with TB, *Mycobacterium* is especially adept at aerosolization, permitting it to be the dominant taxon in aerosol. Besides the enrichment of *Mycobacterium* in aerosols vs. sputum, *Sphingobium* and *Corynebacterium* were enriched, however, their role in TB airborne survival and transmission requires future evaluation.

Our study had limitations. Although the largest to date on this topic that included invasive forms of sampling (bronchoscopy), different findings might result from larger sample sizes. Although the dependencies on parent studies enhanced the feasibility of our work, it also resulted in not all people receiving all procedures. We also only evaluated people with TB; aerosol from people without TB may have different differential taxa.

In summary, PMC captures aerosols which can be used to detect MTBC using WHO-approved molecular tests and sequencing. The taxonomic composition of aerosol differs to that in other respiratory fluids. This work lays a foundation for research on the aeromicrobiome in TB.

## Figures and Tables

**Table 1:** Demographic and clinical characteristics. Data are median (IQR) or n (%)

Characteristic	Participants (n = 16)
*Demographic*	
Age, years	35 (25–46)
Female	9 (56)
Ethnicity	
Black	1 (6)
Mixed ancestry	15 (94)
Smokers	12 (75)
BMI (kg.m^−2^) categories	
Underweight (< 18.5)	12 (75)
Normal (18.5–24.9)	4 (25)
*Clinical*	
HIV	5 (31)
TTP	5 (5, 7)

**Table 2:** Summary of Ultra-based MTBC detection in aerosols, showing Ultra results when applied to the bag and filter of PMC. There was no difference in sputum bacillary load (TTP) and PCR-inhibition across all comparisons. Data are % (n/N) or median (IQR) or mean (± SD).

Ultra result on captured aerosol	Bag n = 13*	Filter n = 14	*p*-value
**Positive Ultra**	54 (7/13)	43 (6/14)	0.568
Sputum TTP	5 (5, 7)	7 (5, 11)	0.392
SPC C_T_	26.25 ±0.82	26.38 ±1.50	0.999
IS1081-IS6110 C_T_	25.66 ±1.29	26.30 ±0.460	0.274
**Negative Ultra**	46 (6/13)	57 (8/14)	-
Sputum TTP	7 (7, 12)	6 (5, 9)	0.161
SPC C_T_	26.25 ±0.575	26.68 ±0.538	0.212
**SPC C_T_**	26.01 ±1.10	26.18 ±0.998	0.675
**SPC C_T_ Ultra-positive vs. Ultra-negative bags**	25.66 ±1.29 vs.	26.42 ±0.711	0.227
**SPC C**_**T**_ **Ultra-positive vs. Ultra-negative filters**	26.30 ±0.460 vs	. 26.09 ±1.294	0.710

* Of the 13 people with both actionable Ultra results from both bag and filter, 4/13 were bag- and filter-positive, 3/13 were only bag-positive, and 2/13 were only filter positive. Abbreviations:

PneumoniaCheck (PMC), days to positivity (TTP), Xpert MTB/RIF Ultra (Ultra), sample processing control (SPC) and cycle threshold (C_T_).

## Data Availability

Data is available on reasonable request. Study protocol and datasets generated in this study maybe requested from the corresponding author.
